# Targeted therapeutic mild hypercapnia after cardiac arrest

**DOI:** 10.1186/s13054-017-1770-6

**Published:** 2017-07-31

**Authors:** Glenn M. Eastwood, Alistair Nichol, Matt P. Wise

**Affiliations:** 1 Department of Intensive Care, Austin Hospital, Melbourne, Victoria Australia; 2 Australia and New Zealand Intensive Care Research Centre, Monash University, Melbourne, Victoria Australia; 3 Department of Intensive Care, Alfred Hospital, Melbourne, Victoria Australia; 4 Irish Critical Care- Clinical Research Network, University College Dublin / St Vincent’s University Hospital, Dublin, Ireland; 5 Adult Critical Care, University Hospital of Wales, Cardiff, UK

We read the paper entitled “Clinical pathophysiology of hypoxic ischemic brain injury after cardiac arrest: a “two-hit” model” by Sekhon et al. published in *Critical Care* [1]. We agree that the clinical pathophysiologic impact of hypoxic ischemic brain injury after cardiac arrest (CA) is multi-factorial and complex. We are concerned, however, that the description of carbon dioxide management fails to address the potential therapeutic role of mild hypercapnia and identify current research directions underway to explicitly investigate targeted mild hypercapnia in the early post-resuscitation period.

Previously, from a large retrospective audit involving >16,000 patients from 125 Australian and New Zealand (ANZ) intensive care units (ICUs) following CA between 2000 and 2011, we found that, compared with normocapnia, hypercapnia was associated with a greater likelihood of discharge home among survivors [2]. More recently, the findings of our prospective phase II multi-centre randomised trial, the CCC trial, showed that targeting therapeutic mild hypercapnia (TTMH) (PaCO_2_ 50-55 mmHg), compared to targeted normocapnia (TN) (PaCO_2_ 35-45 mmHg) was feasible, appeared safe and resulted in attenuation of neuron specific enolase (NSE) release (a biomarker of brain injury) in resuscitated CA patients [3]. While at this stage only hypothesis generating, such findings suggest that mild hypercapnia could have neuro-protective properties during the immediate post-resuscitation phase. If proven to be beneficial, induction of hypercapnia would be an easy to apply intervention at minimal cost to most CA patients.

We agree that the current best evidence indicates that hypocapnia should be avoided, there is uncertainly whether normocapnia or TTMH is the optimal approach in the immediate post-resuscitation phase. There is sufficient uncertainty to justify a definitive trial to evaluate the potential benefit of mild hypercapnia. Indeed, we have now initiated the TAME Cardiac Arrest trial (Clinicaltrials.gov NCT03114033). This phase III multi-centre randomised controlled trial will determine whether TTMH, applied during the early post-resuscitation period, improves neurological outcome of resuscitated adult cardiac arrest patients admitted to the intensive care unit.

## Abbreviations

CA: Cardiac arrest
TN: Targeted normocapnia
TTMH: Targeted therapeutic mild hypercapnia
