# Supplementary material for: Targeted therapeutic mild hypercapnia after cardiac arrest
Source: Crit Care. 2017 Jul 31;21:196. doi: 10.1186/s13054-017-1770-6 (PMC5535289; doi:10.1186/s13054-017-1770-6)
Supplement: Related texts

# Reply: Authors’ response

**Authors:** Mypinder S. Sekhon, Philip N. Ainslie, Donald E. Griesdale

*To the editor:*

We acknowledge Eastwood et al. for their insightful comments to our narrative review entitled “Clinical pathophysiology of hypoxic ischemic brain injury after cardiac arrest: a “two hit” model”. They identify the importance of preventing secondary cerebral injury after cardiac arrest and the crucial role of arterial carbon dioxide induced modulation of cerebral blood flow and oxygen delivery. This effect occurs through the modulation of extracellular pH and is rendered less effective over time. Clearly, episodes of hypocapnia are associated with adverse outcome, stemming from cerebral vasoconstriction, reduced cerebral blood flow and oxygen delivery [1]. Conversely, in a large multicenter observational study, Schneider et. al demonstrated that patients with hypercapnia (PaCO2 > 45 mmHg on one blood gas in the first 24 hours) had higher rates of discharge home among survivors (OR 1.16, 95%CI: 1.03 – 1.32) [2].

Biologically, the phenomenon of “no reflow”, characterized by cerebral vasoconstriction, microvascular thrombi and subsequent cerebral oligemia early after resuscitation is well established [1]. It follows that mild hypercapnia to induce cerebral vasodilation may augment blood flow during this critical period and mitigate secondary injury. We commend Eastwood et al. for undertaking a foundational randomized control trial to investigate the role of mild hypercapnia (PaCO_2_ 50-55 mmHg) after cardiac arrest compared with normocapnia (PaCO_2_ 35-45 mmHg) (TAME trial; Clinicaltrials.gov NCT03114033) and eagerly await the results.

This trial will no doubt provide important insights into the management of post-resuscitative care of cardiac arrest patients. However, it should be noted that there is likely significant within-patient heterogeneity with respect to individual pathophysiology of hypoxic ischemic brain injury [1]. Randomized control trials of single physiological interventions in critical care may fail to account for these nuances. For example, two recent randomized trials of single physiological interventions aimed at mitigating secondary injury after cardiac arrest and traumatic brain injury, specifically targeted temperature management and transfusion thresholds, respectively, were negative [4, 5]. Hence our belief is that we should shift the paradigm to delineating the underlying individualized pathophysiology and establish personalized physiologic resuscitation targets following cardiac arrest. Our research group has recently demonstrated the ability to monitor cerebral autoregulation in real time after cardiac arrest to identify optimal and individualized mean arterial pressure [6]. We acknowledge that it remains unknown if individualized perfusion targets are associated with improved outcome after cardiac arrest. However, this strategy represents an intriguing shift towards personalized physiological resuscitation in the management of this catastrophic and complex disease.
